# PSMA-GCK01: A Generator-Based ^99m^Tc/^188^Re Theranostic Ligand for the Prostate-Specific Membrane Antigen

**DOI:** 10.2967/jnumed.122.264944

**Published:** 2023-07

**Authors:** Jens Cardinale, Frederik L. Giesel, Christina Wensky, Hendrik G. Rathke, Uwe Haberkorn, Clemens Kratochwil

**Affiliations:** 1 Department of Nuclear Medicine, University Hospital Heidelberg, Heidelberg, Germany;; 2 Department of Nuclear Medicine, Medical Faculty and University Hospital Duesseldorf, Heinrich Heine University Duesseldorf, Duesseldorf, Germany; and; 3 Clinical Cooperation Unit Nuclear Medicine, German Cancer Research Center, Heidelberg, Germany

**Keywords:** PSMA, ^188^Re, ^99m^Tc, theranostic, SPECT

## Abstract

Prostate-specific membrane antigen (PSMA) theranostics have been introduced with ^68^Ga and ^177^Lu, the most used radionuclides. However, ^188^Re is a well-known generator-based therapeutic nuclide that completes a theranostic tandem with ^99m^Tc and may offer an interesting alternative to the currently used radionuclides. In the present work, we aimed at the development of a PSMA-targeted ^99m^Tc/^188^Re theranostic tandem. **Methods:** The ligand HYNIC-iPSMA was chosen as the lead structure. Its HYNIC chelator has limitations for ^188^Re labeling and was replaced by mercaptoacetyltriserine to obtain PSMA-GCK01, a precursor for stable ^99m^Tc and ^188^Re labeling. ^99m^Tc-PSMA-GCK01 was used for in vitro evaluation of the ligand and comparison with ^99m^Tc-EDDA/HYNIC-iPSMA. Planar imaging using ^99m^Tc-PSMA-GCK01 and organ biodistribution with ^188^Re-PSMA-GCK01 were performed using LNCaP tumor–bearing mice. Finally, the theranostic tandem was applied for imaging and therapy in 3 prostate cancer patients in compassionate care. **Results:** Efficient radiolabeling of PSMA-GCK01 with both radionuclides was demonstrated. Cell-based assays with ^99m^Tc-PSMA-GCK01 versus ^99m^Tc-EDDA/HYNIC-iPSMA revealed comparable uptake characteristics. Planar imaging and organ distribution revealed good tumor uptake of both ^99m^Tc-PSMA-GCK01 and ^188^Re-PSMA-GCK01 at 1 and 3 h after injection, with low uptake in nontarget organs. In patients, similar distribution patterns were observed for ^99m^Tc-PSMA-GCK01 and ^188^Re-PSMA-GCK01 and in comparison with ^177^Lu-PSMA-617. **Conclusion:** The ligand PSMA-GCK01 labels stably with ^99m^Tc and ^188^Re, both generator-based radionuclides, and thus provides access to on-demand labeling at reasonable costs. Preclinical evaluation of the compounds revealed favorable characteristics of the PSMA-targeted theranostic tandem. This result was confirmed by successful translation into first-in-humans application.

## Visual Abstract

**Figure:**
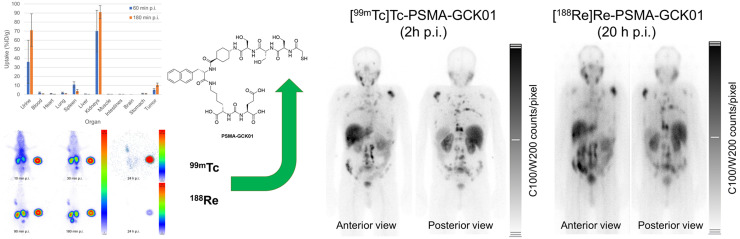

The development of theranostics has dominated recent activities in the field of oncologic nuclear medicine. The most common theranostics are based on so-called matched pairs, in which the diagnostic and therapeutic radiopharmaceuticals are labeled with radionuclides from different elements. Sometimes, even different molecules are used. The only precondition is that both radiopharmaceuticals within the matched pair show a similar biodistribution (1–3).

A combination of nuclides using the same precursor that fulfills this demand is ^99m^Tc with ^188^Re (and ^186^Re) (4). ^99m^Tc is still one of the most widely used radionuclides worldwide, and rhenium (with its isotopes ^186^Re and ^188^Re) is the only element resembling its in vivo chemistry nearly perfectly. Moreover, both ^99m^Tc and ^188^Re are available from radionuclide generators, disclosing potential application in areas without strong nuclear infrastructure. Finally, ^188^Re might help to surpass potential shortages in the supply of ^177^Lu, which may arise from lack of high-flux neutron facilities (5). This renders ^99m^Tc/^188^Re-based theranostic radiopharmaceuticals an attractive combination, in particular for smaller hospitals using only SPECT in their nuclear medicine departments, as well as for application in developing countries (6). Another more current aspect underscores the need for SPECT-based prostate-specific membrane antigen (PSMA) imaging and therefore ^99m^Tc-ligands: the patient selection for recently approved ^177^Lu-PSMA-617 (Pluvicto; Advanced Accelerator Applications) affords confirmation of sufficient PSMA uptake in a preliminary diagnostic scan. For these mandatory diagnostic scans, current PET infrastructure may prove to be the bottleneck—a challenge that might be met by suitable SPECT ligands, including ligands developed primarily for diagnostic purposes, such as ^99m^Tc-MIP-1404 (7) and ^99m^Tc-EDDA/HYNIC-iPSMA (8).

The aim of this work was to develop a ^99m^Tc/^188^Re theranostic tandem targeting PSMA. The ligand HYNIC-iPSMA, which is already in an advanced clinical stage in its technetium-labeled form ^99m^Tc-EDDA/HYNIC-iPSMA, was chosen as the lead structure (8–10). ^99m^Tc is coordinated via the HYNIC chelator in ^99m^Tc-EDDA/HYNIC-iPSMA. However, it is rather unlikely that HYNIC is a suitable chelator for ^188^Re (6). Thus, we replaced the chelator in HYNIC-iPSMA with the more suitable mercaptoacetyltriserine, a classical N_3_S chelator suitable for ^188^Re coordination (Fig. 1) (11*,*^12^). In the following, we provide a summary of our research results thus far, including first-in-humans application under compassionate use.

**FIGURE 1.**
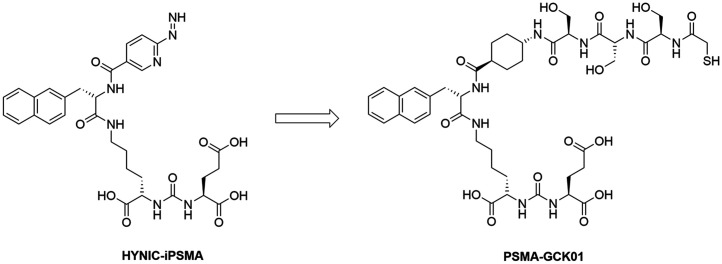
Structures of HYNIC-iPSMA and PSMA-GCK01.

## MATERIALS AND METHODS

### General

Precursor was synthesized using well-known standard methods as described in the supplemental materials (supplemental materials are available at http://jnm.snmjournals.org) (13*,*^14^). The SnCl_2_ for rhenium reduction was trace metal–based and acquired from Merck. The remaining chemicals for tracer synthesis were all of European Pharmacopoeia or *ad injectabilia* grade and acquired from Merck or B. Braun. Waters Sep-Pak Alumina N Light cartridges, Sep-Pak Accell Plus QMA Plus Light cartridges, and Sep-Pak tC18 Plus Light cartridges were purchased from Macherey and Nagel. Dionex OnGuard II Ag cartridges were purchased from Thermo Fischer.

^99m^Tc was obtained from a TekCis generator (Curium) in the form of ^99m^Tc-NaTcO_4_ in 0.9% saline, according to the manufacturer instructions. ^188^Re was obtained in the form of ^188^Re-NaReO_4_ from a ^188^W/^188^Re tandem generator (OnkoBeta) by elution with 10 mL of 0.9% saline (B. Braun). ^99m^Tc-EDDA/HYNIC-iPSMA was produced from commercial kits according to the instructions. The reagent kits were acquired from Telix Pharmaceuticals.

### Chemistry and Radiochemistry

#### ^99m^Tc Labeling of PSMA-GCK01

Phosphate buffer for labeling was prepared from 890 mg of Na_2_PO_4_⋅2H_2_O in 9.5 mL of water for injection and 0.5 mL of 2 M NaOH (pH 11.5–12.0). For ^99m^Tc labeling, 500–800 μL of pertechnetate solution (1.5–2.5 GBq/mL in 0.9% saline) were mixed with 200 μL of phosphate buffer, 100 μL of tris(2-carboxyethyl)phosphine (28.9 mg/mL in phosphate buffer), and a 20-μL precursor solution (1 mg/mL). The resulting mixture (pH 8.0–8.5) was heated at 98°C for 10 min. The mixture was diluted with approximately 1 mL of 0.9% saline and passed through a C18 cartridge (a Sep-Pak tC18 Plus Light cartridge preconditioned with 5 mL of ethanol, followed by 10 mL of water). The product was eluted with 1 mL of 70% ethanol and diluted with 9 mL of phosphate-buffered saline (PBS), prepared from 9 mL of 0.9% saline and 1 mL of phosphate buffer concentrate, both *ad injectabilia* (B. Braun). Finally, the product was passed through a 0.22-μm sterile filter. Samples of the reaction mixture—withdrawn directly after the reaction, after cartridge separation (but before dilution with PBS)—and of the final product formulation were analyzed by reverse-phase high-performance liquid chromatography (HPLC).

#### ^188^Re Labeling of PSMA-GCK01

^188^Re was eluted from the ^188^W/^188^Re tandem generator using 10 mL of 0.9% NaCl. The eluate was postprocessed according to Guhlke et al. (15). Potential tungsten breakthrough was retained on a Sep-Pak Alumina N cartridge. The eluate was dechlorinated using a Dionex OnGuard II Ag cartridge, and the perrhenate was concentrated using a Sep-Pak QMA cartridge, preconditioned with 5 mL of 1 M K_2_CO_3_, followed by 10 mL of deionized water. The perrhenate was eluted from the QMA cartridge using 1 mL of 0.9% NaCl (B. Braun).

A typical ^188^Re-labeling mixture consisted of 120 μL of citrate solution (100 mg/mL), 80 μL of GCK01 precursor solution (1 mg/mL in MeCN/H_2_O 50:50 v/v), 40 μL of 30% ascorbic acid solution (in water), 800 μL of perrhenate in 0.9% NaCl (postprocessed as described earlier at 6–12 GBq), and 48 μL of SnCl_2_ (50 mg/mL in 1 M HCl). The mixture was usually pH 2.0–3.5. The mixture was heated at 96°C for 60 min. After being cooled to ambient temperature, the mixture was neutralized to pH 7.5 using 0.5 M sodium phosphate, heated for an additional 5 min at 96°C, diluted with 1 mL of 0.9% NaCl, and passed through a Sep-Pak tC18 Plus Light cartridge (preconditioned with 5 mL of ethanol and 10 mL of water). The cartridge was washed with 2–3 mL of 0.9% NaCl, and the product was eluted with 1 mL of 70% ethanol. The solution containing the product was diluted 1:9 into PBS, prepared from 9 mL of 0.9% NaCl and 1 mL of sodium phosphate concentrate, both *ad injectabilia* (B. Braun), containing 2% sodium ascorbate solution. The radiochemical yield was determined by division of the isolated product activity by the starting activity. The radiochemical purity was determined by radio-HPLC for the isolated product (after cartridge separation and formulation).

### Preclinical Evaluation

#### In Vitro and Toxicologic Evaluation of the ^99m^Tc-/^188^Re-PSMA-GCK01 Tandem

The evaluation was conducted using well-known standard methods (16*,*^17^). Detailed information is provided in the supplemental materials.

Cellular uptake experiments were conducted in analogy to a previously described procedure (16). A detailed description is provided in the supplemental materials.

#### In Vivo and Organ Distribution Experiments

All animal experiments were conducted in compliance with the current laws of the Federal Republic of Germany (animal license 35-9185.81/G-127/(18)). For in vivo planar imaging and organ distribution experiments, 8-wk-old BALB/c *nu/nu* mice (male) were subcutaneously inoculated in the left shoulder with 6 million LNCaP cells in 50% Matrigel (Corning) in Opti-MEM I medium. The studies were performed when the tumor size reached approximately 1 cm^3^ (8–12 wk after inoculation). Mean body weight was 23 ± 2 g on the day of investigation.

#### In Vivo Planar Imaging

For the in vivo planar imaging, 100 μL of a formulation containing 5–10 MBq of ^99m^Tc-labeled compound in PBS containing ∼0.1 μg precursor (1 nM, 5–10 MBq/nmol) was injected into the tail vein of a LNCaP tumor–bearing mouse (*n* = 1). The animal was anesthetized with isoflurane (AbbVie Deutschland GmbH) and placed prone on a γ-imager (S/C) for planar imaging (using γ-acquisition and GammaVision+ (Biospace Mesures) software). The scan was started directly after administration of the activity, and the mouse was scanned for 10 min. The scan was repeated after 30, 90, and 180 min and 24 h. An activity standard (∼1 MBq of the respective tracer) was prepared in a closed HPLC sample flask and placed next to the animal during all time points of the measurement.

#### Ex Vivo Organ Distribution with ^188^Re-PSMA-GCK01

For ex vivo biodistribution, LNCaP tumor–bearing mice were injected with 100 μL of a PBS formulation containing approximately 1 MBq of ^188^Re-PSMA-GCK01 (∼0.1 μg of precursor or 1 μg of precursor per milliliter). The animals were killed by CO_2_ asphyxiation 1 and 3 h after injection, respectively. Organs of interest were dissected, blotted dry, and weighed, and the radioactivity was determined on a γ-counter (Packard Cobra II; GMI) and calculated as percentage injected dose (%ID) per gram.

### Clinical Imaging and Therapy

After giving written informed consent, 3 patients with metastatic castration-resistant prostate cancer received PSMA-radioligand therapy (RLT) and the related companion diagnostic under compassionate-care regulations. Prospective clinical trial registration is not required for compassionate care that is performed under an individual medical indication. The ethical committee of the University Hospital Heidelberg approved the retrospective evaluation (permission S-732/18). The patients were Gleason score 9 (*n* = 2) or 10 (*n* = 1), and all cases were metastatic to lymph nodes and bone but without visceral lesions All patients had previously received standard androgen-deprivation therapy, abiraterone, prednisolone, docetaxel; 2 patients had additionally received enzalutamide, 1 patient had also received apalutamide, and 2 patients had additionally received cabazitaxel. All cases were BRCA1/2 wild-type and naïve to poly(adenosine diphosphate ribose) polymerase inhibitors, and none of the patients was a promising candidate for ^223^RaCl_2_ (low uptake in bone scan or bulky lymph nodes). One patient had previously received ipilimumab with nivolumab and 2 cycles of ^177^Lu-PSMA-617; the other 2 patients were ^177^Lu-PSMA–naïve.

**FIGURE 2.**
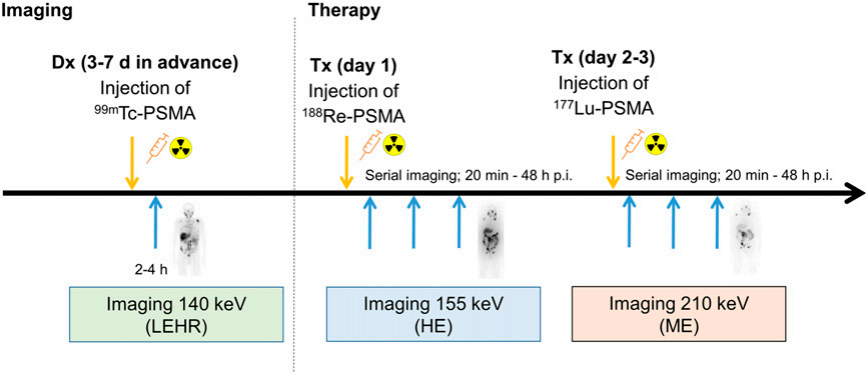
Dual-photopeak imaging for intraindividual comparison of Re-PSMA-GCK01 vs. Lu-PSMA-617 pharmacokinetics. Dx = diagnosis; HE = high-energy; LEHR = low-energy high-resolution; ME = medium-energy; p.i. = after injection; Tx = treatment;

To demonstrate target-positive disease, a diagnostic scan was performed about 1 wk in advance of therapy using 600 MBq of ^99m^Tc-PSMA-GCK01 (molar activity, 75–125 MBq/nmol), and images were acquired 2–4 h after injection with an low-energy, high-resolution collimator at a 140-keV (±10%) photopeak (18 cm/min; E.Cam [Siemens]). At day 1 of therapy, 3.7 GBq of ^188^Re-PSMA-GCK01 (molar activity, 56–112 MBq/nmol) were administered, followed by serial planar scans (20 min to 48 h as clinically available) centered at the 155-keV (±10%) photopeak but with a high-energy collimator because of downscatter of up to 2.12-MeV bremsstrahlung (E.Cam [Siemens], 18 cm/min). After 1–2 d (∼2–3 physical half-lives of ^188^Re), when septum penetration of bremsstrahlung became negligible, 3.7 GBq of ^177^Lu-PSMA-617 were injected and serial images were acquired with a medium-energy collimator; to avoid cross-talk with the primary 155-keV photons of ^188^Re, only the upper photopeak of ^177^Lu at 210 keV (±10%) was used. Dual-photopeak imaging within such a short time enables intraindividual comparison of 2 therapeutic ligands, even though relevant treatment-related effects have yet to be considered. The timeline of imaging time points is illustrated in Figure 2. However, under these circumstances, no scatter-subtraction techniques could be applied to obtain sufficient quantitative data.

## RESULTS

### Chemistry and Radiochemistry

#### Precursor

The identity of the precursor was confirmed by HPLC–mass spectrometry: *m/z* = 977.386 (calculated *m/z* ([M+H^+^]^+^) = 977.392) and *m/z* = 999.367 (calculated *m/z* ([M+Na^+^]^+^) = 999.374). The purity was analyzed by HPLC and was more than 95%. The only detectable impurity was the oxidized disulfide. Details are provided in Supplemental Figures 1–3.

#### ^99m^Tc-PSMA-GCK01

The radiosynthesis of ^99m^Tc-PSMA-GCK01 reliably delivered the product in radiochemical yields of 81% ± 3% and purities above 97.8% ± 0.7% after cartridge separation (*n* = 8). Molar activity was in the range of 75–125 MBq/nmol. Residual tris(2-carboxyethyl)phosphine was removed quantitatively by cartridge separation. No signs of degradation were observed over a period of 7 h. Details are provided in Supplemental Figures 4 and 5.

#### ^188^Re-PSMA-GCK01

After ^188^Re postprocessing, approximately 79% ± 6% of the total activity was retrieved in 1 mL of saline without detectable ^188^W breakthrough (*n* = 9). Subsequent labeling yielded ^188^Re-PSMA-GCK01 in radiochemical yields of 78% ± 3% and with a radiochemical purity of more than 96% ± 3% (*n* = 6). Molar activity was in the range of 56–112 MBq/nmol. Only minor signs of degradation were observed after 3 h (∼3% degradation of radiochemical purity). Details are provided in Supplemental Figure 6.

### Preclinical Evaluation

#### In Vitro Evaluation

The binding characteristics of PSMA-GCK01 and HYNIC-iPSMA were evaluated with the respective ^99m^Tc-labeled tracers; the results are summarized in Table 1. ^99m^Tc-PSMA-GCK01 showed plasma protein binding of 98%. The radiochemical purity of the free fraction showed no signs of degradation over 4 h (by repetitive HPLC measurements; Supplemental Fig. 7).

**TABLE 1.** Binding Characteristics of ^99m^Tc-EDDA/HYNIC-iPSMA and ^99m^Tc-PSMA-GCK01

Substance	Uptake (%AD/10^6^ cells)	Unspecific uptake (%AD/10^6^ cells)	Specific uptake (%AD/10^6^ cells)	K_i_ (nm)
^99m^Tc-EDDA/HYNIC-iPSMA (reference compound)	20.3 ± 0.3	1.64 ± 0.07	18.7 ± 0.3	38
^99m^Tc-PSMA-GCK01	19.6 ± 4.8	1.2 ± 0.6	18.4 ± 4.2	26

%AD = Applied dose; K_i_ = inhibition constant.

#### In Vivo and Organ Distribution Experiments

**FIGURE 3.**
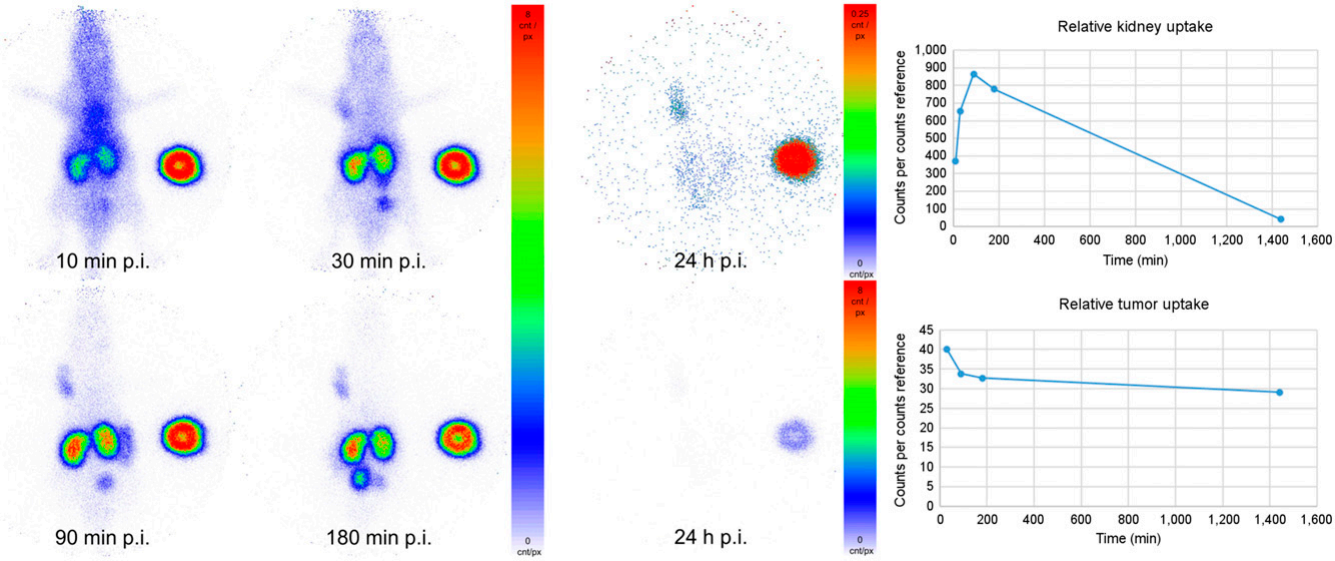
Planar imaging of ^99m^Tc-PSMA-GCK01 in LNCaP tumor–bearing mouse. Red spot at right side is internal standard. p.i. = after injection.

The images acquired by in vivo planar imaging are shown in Figure 3. Region-of-interest analysis and standardization on the internal standard provided a rough estimation of the observed uptake values of ^99m^Tc-PSMA-GCK01.

**FIGURE 4.**
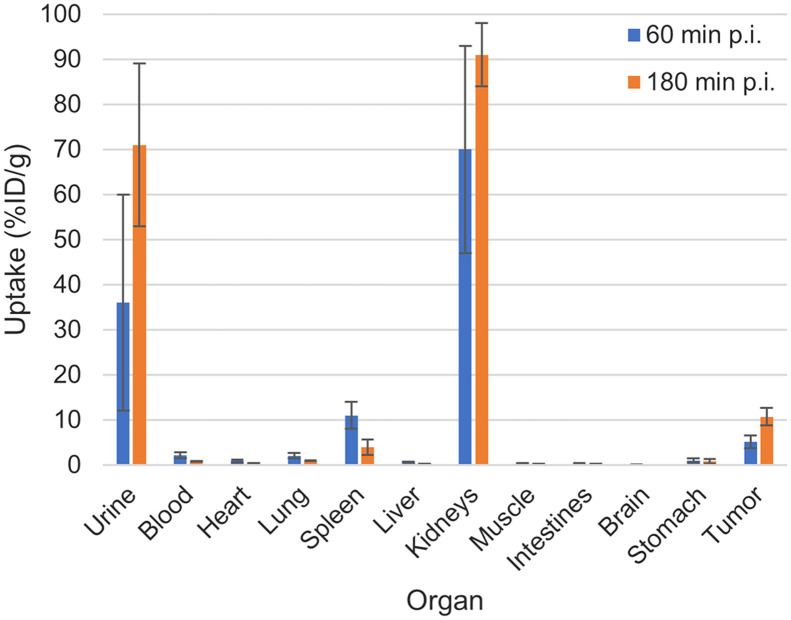
Organ distribution of approximately 1 MBq of ^188^Re-PSMA-GCK01 in LNCaP tumor–bearing mice (3 per time point). Exact values are provided in Supplemental Table 1. p.i. = after injection.

The results of the organ distribution of ^188^Re-PSMA-GCK01 are depicted in Figure 4. The tumor uptake of the ligand is approximately 5 %ID/g at 1 h after injection, rising to approximately 11 %ID/g at 3 h after injection. In addition, the ligand showed an uptake of 70 %ID/g (1 h after injection) and 91 %ID/g (3 h after injection) in kidneys and 11 %ID/g (1 h after injection) and approximately 4 %ID/g (3 h after injection) in the spleen. Moreover, renal excretion is reflected by urine uptake of 36 %ID/g (1 h after injection) and 71 %ID/g (3 h after injection). All further organs showed only minor tracer uptake. Detailed results are provided in the supplemental materials.

#### Toxicologic Investigation

No test article–related mortality was observed. No differences in organ weights or macroscopic observations were seen at terminal or recovery euthanasia.

### Clinical Imaging and Therapy

**FIGURE 5.**
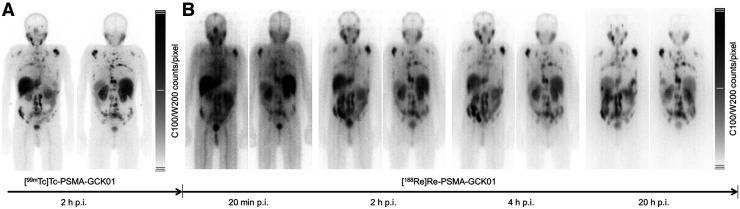
^188^Re-GCK01 photopeak (sequential imaging). p.i. = after injection.

In the 3 compassionate-care patients, no acute adverse events were observed after injection of ^99m^Tc-PSMA-GCK01 or ^188^Re-PSMA-GCK01. Visually, the gross biodistribution of diagnostic ^99m^Tc-PSMA-GCK01 was similar to that of all other low-molecular-weight scintigraphic PSMA ligands that had been developed previously (Fig. 5A), with combined renal and hepatointestinal clearance, nontarget accumulation in salivary glands, and a low perfusion-dependent background in the remaining organs (8*,*^9^*,*^18^*,*^19^). Monoenergetic, 140-keV, pure γ-emitting ^99m^Tc-PSMA-GCK01 was imaged with a low-energy, high-resolution collimator, but ^188^Re-PSMA-GCK01 had to be measured with a high-energy collimator to cope with the high level of scatter and bremsstrahlung (up to 2.12 MeV) in relation to the only 15% coemission probability of 155-keV photons. Considering these limitations, the tagged radionuclide had no obvious influence on biodistribution 2–4 h after injection (Fig. 5). Already 20 min after injection, the intensity of tumor targeting exceeded the intravascular blood pool and delineation of the bladder demonstrated moderate clearance kinetics. Late images beyond 20 h after injection demonstrated prolonged trapping in tumor lesions, additional hepatobiliary clearance into the intestine, and low residual uptake in other organs (Fig. 5B); thus, to some degree, PSMA-GCK01 has tumor accumulation and excretion kinetics similar to those of other Glu-urea–based PSMA ligands, such as MIP-1095, PSMA-617, or PSMA-I&T (20–22).

**FIGURE 6.**
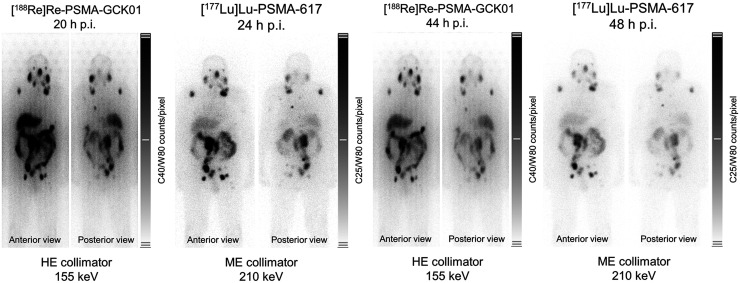
Intraindividual comparison of 3.7 GBq of Lu-PSMA-617 and 3.7 GBq of Re-PSMA-GCK01 at 20–24 and 44–48 h after injection, respectively. HE = high-energy; ME = medium-energy; p.i. = after injection.

Using the distinct photopeaks of ^188^Re at 155 keV, followed 48 h later by ^177^Lu imaged at 210 keV, an intraindividual comparison between ^188^Re-PSMA-GCK01 and ^177^Lu-PSMA-617 within a 2-d interval is demonstrated in Figure 6. Although tumor targeting is almost equal at 20–24 and 44–48 h after injection, PSMA-GCK01 initially demonstrates a higher liver-to-kidney uptake ratio, which translates into better delineation of the intestine 48 h after injection, possibly implying a slight shift from renal to hepatointestinal clearance for PSMA-GCK01 compared with PSMA-617.

## DISCUSSION

The focus of our investigation was the development of a PSMA ligand suitable for ^99m^Tc and ^188^Re labeling based on the HYNIC-iPSMA lead structure. Because the suitability of HYNIC for ^188^Re labeling has been controversial (6), we replaced the HYNIC unit with a similar-in-size spacer, linked to the mercaptoacetyltriserine sequence as a favorable chelator for radiolabeling with both ^99m^Tc and ^188^Re (Fig. 1). The precursor synthesis was obtained in high purity of more than 95%. The only detectable impurity was the oxidized disulfide derivate (Supplemental Figs. 2 and 3), which is reduced to the desired precursor under the reductive labeling conditions.

Technetium labeling using tris(2-carboxyethyl)phosphine as the reducing agent delivered the product ^99m^Tc-PSMA-GCK01 in high and reproducible yields and purities. In general, the radiochemical purity would allow direct application of the product mixture containing the tracer. However, for clinical formulation, we applied cartridge separation of the tracer to remove residual tris(2-carboxyethyl)phosphine, which was confirmed by HPLC analysis (Supplemental Fig. 5). The reducing agent may impose a potential limitation. For broader application, a kit should be developed using the more commonly applied SnCl_2_ as the reducing agent.

In the case of ^188^Re labeling, the desired ^188^Re-PSMA-GCK01 was produced in yields of approximately 75% (isolated after synthesis and purification) using relatively harsh conditions (low pH). The results agree well with previous reports on such reactions (23). However, under these conditions, the product is formed in 2 stereoisomers (Supplemental Fig. 6). We eventually converted the undesired isomer by terminal pH elevation to pH 7.0–7.5 and a short additional heating period. In the case of ^99m^Tc labeling, the pH was sufficiently high to suppress formation of the isomer. Thus, because no isomer formation was observed in the stable plasma of ^99m^Tc-PSMA-GCK01, we do not consider the potential isomer formation to be a drawback.

For the preclinical evaluation, we first compared our ligand ^99m^Tc-PSMA-GCK01 with ^99m^Tc-EDDA/HYNIC-iPSMA in a cell-based assay. Both compounds showed a comparable and specific uptake in LNCaP cells. A further displacement experiment with ^99m^Tc-PSMA-GCK01 also revealed a comparable inhibition constant of 26 nM (vs. 38 nM for ^99m^Tc-EDDA/HYNIC-iPSMA). The ligand showed relatively high plasma protein binding of 98%. However, for other PSMA ligands, it is known that high plasma protein binding does not necessarily impose a problem for clinical application. Recently, even dedicated albumin-binding motifs have been suggested as an improvement when PSMA ligands should be labeled with long physical half-life nuclides (24*,*^25^). More importantly, the free fraction in plasma did not show signs of decomposition over a period of 4 h. Unfortunately, we were not able to confirm this for the rhenium theranostic tandem ^188^Re-PSMA-GCK01 because of an insufficient count rate (only 15.5% probability of 155-keV emissions) in the respective HPLC samples. Hence, we decided to evaluate our ligands in a suitable animal model. In planar imaging with ^99m^Tc-PSMA-GCK01, the ligand presents promising tumor uptake and retention in the LNCaP xenotransplant and moderate renal clearance, according to semiquantitative region-of-interest analysis. Quantitative data were obtained by further organ distribution experiments with the rhenium analog ^188^Re-PSMA-GCK01. As expected, the results reflected the planar imaging quite well. Already at 1 h after injection, we observed intense tumor uptake, reaching approximately 11 %ID/g at 3 h after injection. In mouse-only renal clearance, minimal uptake in the liver was observed. However, it was already reported for PSMA-617 that renal versus hepatointestinal clearance is not comparable between animal studies and human application (14*,*^26^).

Preclinically, we achieved our goal to develop a promising PSMA ligand for labeling with both ^99m^Tc and ^188^Re for theranostic application. Potential limitations of the current preclinical study are the lack of a late time point (e.g., 24 or 48 h) in organ distribution and the lack of a histopathologic evaluation of eventual radiation-induced kidney toxicity from ^188^Re-PSMA-GCK01 in mice. However, a dedicated clinical dosimetry study is already in preparation (including initial extrapolation from ^99m^Tc-PSMA-GCK01 to ^188^Re-PSMA-GCK01) and will probably be more predictive than the mouse-to-human extrapolation that is otherwise needed for nonradioactive therapies. Another potential limitation is the lack of ^99m^Tc-PSMA-GCK01 organ distribution data. We considered these data to be facultative because the analogy of ^99m^Tc/^188^Re tandem radiopharmaceuticals is widely accepted (4). Consequently, we preferred to reduce our demand for laboratory animals.

Toxicologic investigation of PSMA-GCK01 according to the current Organisation for Economic Co-operation and Development guideline was ordered by a third-party preclinical research organization and revealed no toxicologic effect up to 2 mg/kg in mice. On the basis of these data, we conclude that PSMA-GCK01 has a good safety profile and that application of up to a 2 μg/kg dose of PSMA-GCK01 in humans will likely be well tolerated.

During compassionate use, the promising tumor targeting and acceptable fast clearance kinetics were confirmed in humans. Dual-photopeak imaging enabled intraindividual comparison with the current standard-of-reference compound ^177^Lu-PSMA-617. Our preliminary investigation suggests that ^188^Re-PSMA-GCK01 and ^177^Lu-PSMA-617 share the combined renal and hepatointestinal clearance route and a relatively similar biodistribution between 2 and 20 h after injection. However, using a double-isotope imaging protocol, we could not yet approximate a quantitatively reliable treatment dosimetry. Nevertheless, the ligands offer some promising benefits.

One of these benefits is that the availability of a completely generator-based theranostic tandem to available ligands will help facilitate PSMA-RLT, particularly in countries or regions with a less developed nuclear medicine infrastructure, and may help reduce the cost of PSMA-RLT. Another benefit is that ^188(/186)^Re-PSMA-RLT might amend ^177^Lu-PSMA-RLT in a mixed nuclide therapy, with the potential of offering an additional mean β-emission energy profile (^188^Re, 765 keV; ^186^Re, 347 keV; ^177^Lu, 133 keV) and improving therapy of bulk lesions (27*,*^28^). A third benefit is that shorter-half-life and lower-energy γ-emissions (^188^Re, 16.9 h and 155 keV [15%]; ^186^Re, 3.7 d and 137 keV [9%]; and ^177^Lu, 6.7 d and 113 keV [6%] and 208 keV [10%], respectively) may enable therapy in an outpatient setting, eventually helping to circumvent the expected bottleneck in bed capacity of existing nuclear medicine departments after ^177^Lu-PSMA-617 (half-life, 6.7 d) approval (29).

In summary, we consider the tandem PSMA ligands ^99m^Tc-/^188^Re-PSMA-GCK01 to form a versatile and promising supplement in the available PSMA ligand landscape. In particular, the broad availability of different therapeutic nuclides may lead to interesting synergetic effects that cannot yet be predicted. A phase 1 and 2 clinical trial with the ligands, including a dosimetry study, is in preparation. The additional potential that may arise from the ligand ^186^Re-GCK01 is still to be disclosed.

## CONCLUSION

PSMA-GCK01 is characterized by robust labeling with both radionuclides of the ^99m^Tc-/^188^Re-PSMA-GCK01 theranostic tandem; hence, they can be produced in high radiochemical yields using standard methodologies. Preliminary experiences in patients with metastatic castration-resistant prostate cancer were promising. Thus, further investigation of the ^99m^Tc-/^188^Re-PSMA-GCK01 tandem in a prospective phase 1 trial has already been initiated.

## DISCLOSURE

This project was supported by a research grant from Telix Pharmaceuticals. The toxicologic study at Agilex Biolabs was sponsored by Telix Pharmaceuticals. Frederik Giesel is an advisor at ABX Radiopharmaceuticals, SOFIE Biosciences, Telix Pharmaceuticals, and α-Fusion. Frederik Giesel, Clemens Kratochwil, Uwe Haberkorn, and Jens Cardinale hold a patent application on PSMA-GCK01. No other potential conflict of interest relevant to this article was reported.

## Notes

KEY POINTS **QUESTION:** Can we prepare a PSMA ligand suitable for ^99m^Tc and ^188^Re labeling with HYNIC-iPSMA as the lead structure? **PERTINENT FINDINGS:** An exchange of the chelator moiety to mercaptoacetyltriserine yielded PSMA-GCK01, a ligand suitable for ^99m^Tc and ^188^Re labeling that maintains the good binding characteristics of HYNIC-iPSMA. **IMPLICATIONS FOR PATIENT CARE:** PSMA-GCK01 offers a suitable platform for the decentralized production of the theranostic tandem for prostate cancer patients. In particular, it provides the basis for diagnosis of prostate cancer in smaller nuclear medical centers limited to SPECT, and it may help to circumvent potential bottlenecks in the ^177^Lu supply chain.
